# Paradoxical Embolic Stroke via Pulmonary Arteriovenous Malformation Complicated by Bubble-Study-Induced Non-ST-Segment Elevation Myocardial Infarction

**DOI:** 10.7759/cureus.111048

**Published:** 2026-06-17

**Authors:** Dustin Lee, Daniel Philipson, Celso Diaz

**Affiliations:** 1 Cardiology, University of California, Los Angeles, Los Angeles, USA

**Keywords:** cerebro-vascular accident (stroke), echocardiogram with bubble study, myocardial infarction, paradoxical embolus, pulmonary avm

## Abstract

Pulmonary arteriovenous malformations (PAVMs) are uncommon vascular anomalies that predispose patients to paradoxical embolic phenomena, including stroke. Contrast echocardiography is a key diagnostic tool, but complications are rarely reported. We describe a 44-year-old woman with no significant past medical history who presented with acute ischemic stroke involving the right superior parietal lobe. Initial workup with echocardiography and bubble study demonstrated right-to-left shunting, considered to be of intracardiac origin, typically a patent foramen ovale. However, this was complicated by acute chest pain and troponin elevation consistent with non-ST-segment elevation myocardial infarction (NSTEMI). Coronary angiography revealed angiographically normal coronary arteries. Chest CT subsequently identified a large PAVM, and the patient underwent successful transcatheter occlusion and coil embolization. This case highlights the importance of considering PAVMs in cryptogenic stroke evaluation and underscores the potential for contrast echocardiography to rarely provoke coronary ischemia, even in the absence of obstructive coronary artery disease.

## Introduction

Paradoxical embolism is most commonly associated with patent foramen ovale (PFO), but extracardiac shunts such as pulmonary arteriovenous malformations (PAVMs) can also cause systemic embolization [1,2]. PAVMs are direct connections between pulmonary arteries and veins, bypassing the capillary bed. While often asymptomatic, they can result in hypoxemia, brain abscess, ischemic stroke, or coronary ischemia.

Contrast echocardiography is an invaluable diagnostic modality in suspected right-to-left shunt and is generally considered safe. Early right-to-left shunting (less than five cardiac cycles) usually indicates an intracardiac shunt and late shunting (more than five cardiac cycles) suggests an intrapulmonary shunt. We report a unique case of paradoxical embolic stroke from PAVMs, complicated by bubble-study-induced myocardial infarction despite angiographically normal coronary arteries.

## Case presentation

A 44-year-old woman with no significant past medical history presented to the emergency department with sudden-onset dysarthria. She had no cardiovascular risk factors. On arrival, her blood pressure was 159/90 mmHg, heart rate 66 bpm, and oxygen saturation 96% on room air. Symptoms had resolved by the time the patient arrived at the emergency room (ER) and neurological exam was normal.

Brain MRI revealed acute right superior parietal lobe infarction. MR angiography of the head and neck showed no significant carotid or vertebral stenosis. Transthoracic echocardiography with agitated saline contrast demonstrated borderline early right-to-left shunting (Figure 1), suspicious for intracardiac shunt, typically a patent foramen ovale (PFO).

**Figure 1:**
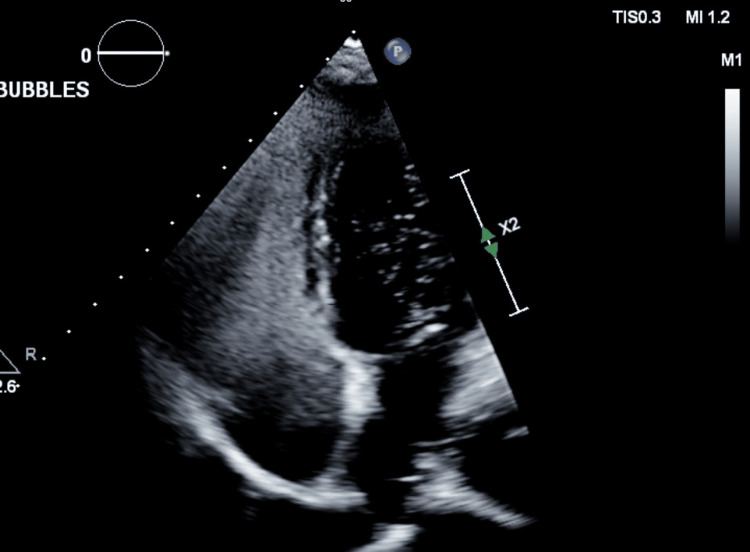
Transthoracic echocardiogram with bubble study showed moderate, borderline early, right-to-left shunting (<5 cardiac cycles), suspicious for intracardiac shunt.

During the study, the patient developed acute substernal chest pain. No acute ischemic ECG changes, but troponin became elevated and peaked at 1650 ng/L. Echocardiogram showed no regional wall motion abnormalities. Subsequent coronary angiography revealed normal coronary arteries, consistent with suspected bubble-study-induced non-ST-segment elevation myocardial infarction (NSTEMI), likely from coronary microvascular embolization of contrast bubbles.

Transesophageal echocardiography confirmed borderline early right-to-left shunting, but could not directly visualize PFO. She was recommended for PFO closure. During this time, she concurrently underwent evaluation for elevated liver enzymes and was incidentally was found to have a suspected right middle lobe PAVM on MR cholangiopancreatogram. Chest CT angiography confirmed the large PAVM (Figure 2). Clinical evaluation indicated a low likelihood of hereditary hemorrhagic telangiectasia (HHT) given no other associated findings.

**Figure 2:**
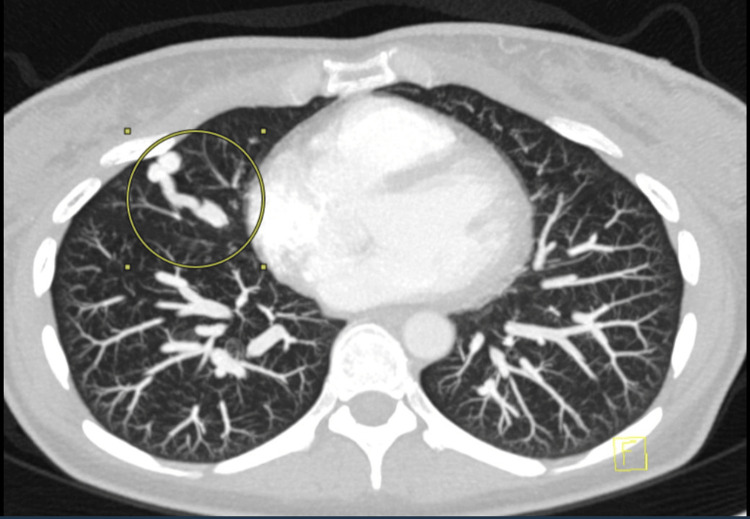
CT Angiogram showing large right middle lobe pulmonary arteriovenous malformation, with feeding artery measuring 4 mm in diameter.

She was planned for a PFO closure and PAVM occlusion. Her PAVM was confirmed on pulmonary arteriogram (Figure 3) and successfully occluded with an Amplatzer Vascular Plug device (Abbott Laboratories, Abbott Park, USA) and coil embolization (Figure 4). Her interatrial septum was interrogated thoroughly, showing no PFO. Repeat bubble study after PAVM occlusion via intracardiac echocardiogram showed no residual right-to-left shunt.

**Figure 3:**
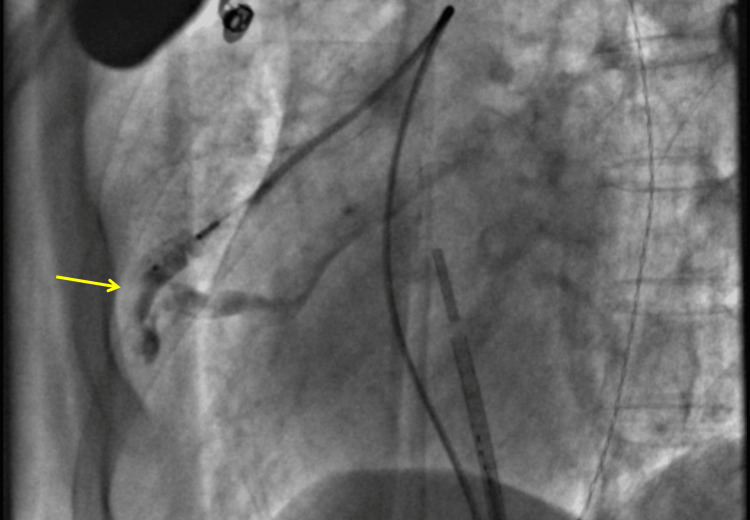
Pulmonary arteriogram showing large pulmonary arteriovenous malformation (AVM).

**Figure 4:**
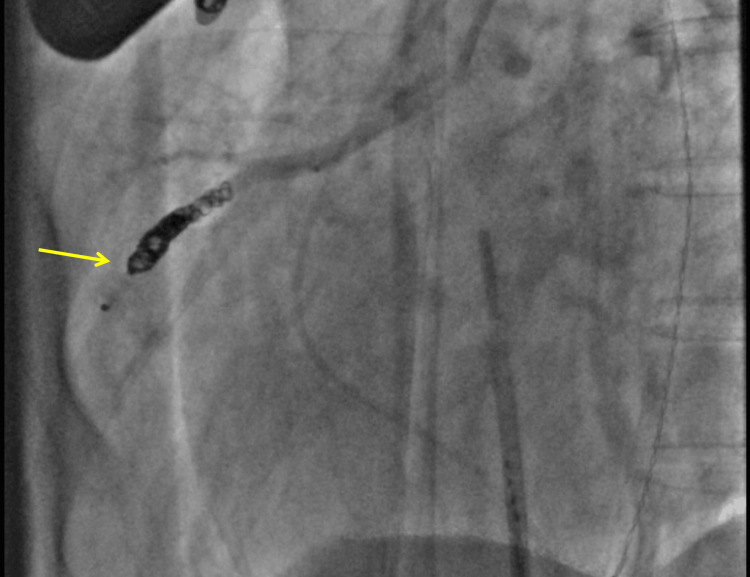
Pulmonary arteriovenous malformation (AVM) status post occluder device and coiling with no residual shunting.

At three-month follow-up, the patient demonstrated no residual neurologic symptoms. Repeat transthoracic echocardiography with bubble study showed no shunting and she had no cardiac symptoms.

## Discussion

PAVMs are abnormal direct communications between the pulmonary arterial and venous systems that bypass the normal pulmonary capillary bed. The pulmonary capillary network ordinarily acts as a mechanical filter, trapping particles before they can reach the systemic circulation. When this filtration is circumvented, embolic material - including thrombi, bacteria, and gas bubbles - can pass directly into the left heart and systemic arterial system, reaching the cerebral, coronary, and other end-organ circulations [1,2].

The majority of PAVMs (approximately 70%-80%) are associated with HHT, an autosomal dominant condition caused by mutations, most commonly in ENG (endoglin) or ACVRL1 (activin receptor-like kinase 1), both encoding proteins involved in transforming growth factor-beta (TGF-β) signaling critical to vascular development [3,4]. PAVMs may also occur sporadically or in association with hepatic cirrhosis, trauma, or connective tissue disorders.

Clinically, small PAVMs may be asymptomatic and discovered incidentally, while larger lesions can produce dyspnea, hypoxemia, and platypnea-orthodeoxia. Neurological sequelae of paradoxical embolism - including ischemic stroke, transient ischemic attack (TIA), and brain abscess - are among the most feared complications; evidence suggests that up to 50% of patients with untreated PAVMs will develop disabling or fatal complications over their lifetime [5,6]. Any young patient presenting with cryptogenic stroke or brain abscess should therefore raise suspicion for an underlying PAVM, as the bypassed pulmonary capillary bed also fails to filter bacteremic emboli.

Diagnosis typically begins with chest imaging. Contrast-enhanced CT of the chest is the modality of choice, allowing precise identification of feeding artery diameter and lesion complexity [6]. Contrast transthoracic echocardiography with agitated saline plays a complementary role in distinguishing intracardiac from extracardiac shunts. In PFO, microbubbles appear in the left heart within three to four cardiac cycles following right-heart opacification; in PAVM-mediated shunting, their appearance is characteristically delayed until after the fourth or fifth cardiac cycle, reflecting transit time through the pulmonary vasculature [7,8]. This timing distinction, demonstrated in this case, is a key diagnostic clue that should prompt dedicated chest imaging rather than empiric closure of a presumed intracardiac defect. It bears noting that this criterion is not absolute - overlap exists - and clinical context and CT confirmation remain essential [8].

This case also underscores a rare but clinically significant complication of bubble contrast echocardiography. When a significant right-to-left shunt is present, microbubbles can bypass the pulmonary circulation, enter the systemic arterial system, and lodge in the coronary microvasculature. The patient's acute chest pain and troponin elevation - in the absence of obstructive coronary artery disease - strongly suggest contrast-induced coronary microembolization as the mechanism of NSTEMI. This mechanism is supported by prior reports of PAVM-associated angina and myocardial infarction via paradoxical embolism [9]. Cases of bubble-study-related ischemic complications remain exceedingly rare in the literature [10]. Clinical vigilance during and after bubble studies is warranted in patients suspected of harboring large right-to-left shunts.

Definitive management of PAVMs is transcatheter embolization, recommended for all lesions with a feeding artery diameter of 3 mm or greater, given the established risk of paradoxical embolism at this threshold [11]. Coil or vascular plug deployment achieves high technical success rates and meaningfully reduces the risk of recurrent stroke, TIA, and brain abscess [11,12]. Surgical resection is now rarely required given advances in catheter-based techniques. This patient's recovery following coil embolization is consistent with favorable outcomes reported in the literature. Long-term surveillance remains essential, as recanalization of embolized lesions and development of de novo PAVMs - particularly in patients with HHT - can occur over time, with recanalization rates reported between 3% and 49% [13].

## Conclusions

This case demonstrates paradoxical embolic stroke caused by PAVMs, with the unusual complication of bubble-study-induced NSTEMI due to coronary microembolization. Clinicians should remain vigilant for PAVMs when bubble echocardiography reveals delayed shunting, and should be aware that contrast studies, though safe in most cases, may rarely provoke myocardial ischemia in patients with right-to-left shunts.
